# Systematic review of burnout among healthcare providers in sub-Saharan Africa

**DOI:** 10.1186/s12889-019-7566-7

**Published:** 2019-09-11

**Authors:** Benyam W. Dubale, Lauren E. Friedman, Zeina Chemali, John W. Denninger, Darshan H. Mehta, Atalay Alem, Gregory L. Fricchione, Michelle L. Dossett, Bizu Gelaye

**Affiliations:** 1 Department of Psychiatry, Addis Ababa University, Addis Ababa, Ethiopia; 2 Department of Epidemiology, Harvard T.H. Chan School of Public Health, 677 Huntington Ave, Kresge 505, Boston, MA 02115 USA; 3 The Chester M. Pierce, M.D. Division of Global Psychiatry, Department of Psychiatry, Massachusetts General Hospital, Boston, MA USA; 4 Benson Henry Institute for Mind Body Medicine at Massachusetts General Hospital, Harvard Medical School, Boston, MA USA

**Keywords:** Burnout, Sub-Saharan Africa, Health personnel

## Abstract

**Background:**

Burnout is characterized by physical and emotional exhaustion from long-term exposure to emotionally demanding work. Burnout affects interpersonal skills, job performance, career satisfaction, and psychological health. However, little is known about the burden of burnout among healthcare providers in sub-Saharan Africa.

**Methods:**

Relevant articles were identified through a systematic review of PubMed, Web of Science (Thomson Reuters), and PsycINFO (EBSCO). Studies were selected for inclusion if they examined a quantitative measure of burnout among healthcare providers in sub-Saharan Africa.

**Results:**

A total of 65 articles met our inclusion criteria for this systematic review. Previous studies have examined burnout in sub-Saharan Africa among physicians (*N* = 12 articles), nurses (*N* = 26), combined populations of healthcare providers (*N* = 18), midwives (*N* = 2), and medical or nursing students (*N* = 7). The majority of studies assessed burnout using the Maslach Burnout Inventory. The highest levels of burnout were reported among nurses, although all healthcare providers showed high burnout. Burnout among healthcare providers is associated with their work environments, interpersonal and professional conflicts, emotional distress, and low social support.

**Conclusions:**

Available studies on this topic are limited by several methodological challenges. More rigorously designed epidemiologic studies of burnout among healthcare providers are warranted. Health infrastructure improvements will eventually be essential, though difficult to achieve, in under-resourced settings. Programs aimed at raising awareness and coping with burnout symptoms through stress management and resilience enhancement trainings are also needed.

**Electronic supplementary material:**

The online version of this article (10.1186/s12889-019-7566-7) contains supplementary material, which is available to authorized users.

## Introduction

Burnout is a psychological syndrome involving emotional exhaustion, feelings of helplessness, depersonalization, negative attitudes towards work and life, and reduced personal accomplishment [1]. The prevalence of burnout in high-income countries among the general working population has been reported to range between 13 and 27% [2, 3]. However, healthcare providers have been described as a high-risk population for experiencing burnout [4–6], and the prevalence of burnout among healthcare providers has been increasing in recent years [7]. The prevalence among physicians is reported to be as high as 70% [8] and nearly 50% among nurses [6, 9, 10]. Studies conducted in the United States show 54% of physicians [7], 35% of hospital nurses [11], and 35.2% of medical students reported burnout [12]. Similar rates of burnout among healthcare providers have been reported in other high-income countries [13–15].

Burnout is of great public health concern due to its physical health consequences including aches, digestive upset, and poor quality of life [12, 16–18]. Furthermore, burnout is highly comorbid with a myriad of psychiatric disorders including depression [19, 20], anxiety [21], substance abuse [19, 22], and suicidality [12, 23] among healthcare providers. In addition to self-reported health outcomes, burnout is associated with hypothalamus-pituitary-adrenal axis dysregulation [24–26], inflammatory responses [27, 28], and increased allostatic load [29, 30]. It has been reported that individuals with occupational burnout exhibit changes in the brain, such as reduction in gray matter volume of the anterior cingulate, caudate and putamen [31]. In addition, occupational burnout has also been associated with a reduced ability to downregulate emotional stressors, altered functioning of the limbic networks [32], and changes in subcortical volume [33]. Studies have shown that physicians with burnout are more likely to report career dissatisfaction and intention to leave the medical profession [34]. Lastly, burnout among healthcare providers has been associated with increased self-reported errors, reduction in time devoted to providing clinical care, and higher mortality rates [35, 36]. In summary, burnout among healthcare providers has profound personal and professional consequences, impacting the quality of patient care and functionality of healthcare systems [37].

Furthermore, appallingly little is known about the collective burden of burnout and its effects on healthcare providers in low- and middle-income countries [38]. Few studies in low- and middle- income countries have reported burnout among healthcare providers including in China [39, 40], Brazil [41], and Egypt [42, 43]. Additionally, there has been an exodus of physicians from sub-Saharan Africa due to the global labor market [44, 45]. In 2015, about 6% of all international medical graduates in the US workforce were from sub-Saharan Africa [46]. Moreover, in half of the countries in sub-Saharan Africa, more than 30% of physicians trained locally have migrated to high-income countries [47]. This has resulted in shortages of healthcare providers in sub-Saharan Africa, and a higher risk of burnout among those who remain to care for a disproportionally greater number of acutely ill patients [47]. Similar migrations from other low- and middle-income countries [48, 49] and from rural areas of high-income countries [50], have led to a scarcity of healthcare providers to care for patients. The remaining healthcare providers have increased responsibility to care for patients and a high risk of burnout. In view of these circumstances, we conducted a systematic literature review to examine the burden of burnout among healthcare providers in sub-Saharan Africa. We were specifically interested in how the construct of burnout was assessed, which healthcare sectors were included, and any interventions that were evaluated. This review is also intended to set the stage for subsequent contributions aimed at reducing the burden of burnout among healthcare providers in sub-Saharan Africa. Effective interventions will need to identify and address individual and structural barriers contributing to burnout among healthcare providers.

## Methods

This systematic review was conducted according to Preferred Reporting Items for Systematic Review and Meta-Analyses (PRISMA) guidelines [51] (Additional file 1: Table S1).

### Study selection and criteria for inclusion

In PubMed, Web of Science (Thomson Reuters), and PsycINFO (EBSCO), we identified studies using search terms for burnout and sub-Saharan African countries (Additional file 1: Table S2). Search terms included all sub-Saharan African countries. All articles published prior to February 14, 2019 were eligible for inclusion. We only included articles available in English. Based on the title and abstract review of all articles, we rejected any articles that were not relevant or did not meet the study criteria. Studies were selected for inclusion if (1) they examined a quantitative measure of burnout, (2) the study population was healthcare providers, and (3) the study was conducted in a sub-Saharan African country. Healthcare providers included physicians, nurses, medical or nursing students, midwives, and other hospital workers. Studies were excluded for (1) not including a quantitative measure of burnout, (2) not measured in healthcare providers, or (3) not conducted in sub-Saharan Africa.

Full texts of articles examining populations of healthcare providers were reviewed. Reference sections of included articles were also reviewed for additional relevant studies. A companion article examines burnout among healthcare providers in the Middle East and Northern Africa (Chemali et al, *under review*).

### Data extraction and quality assessment

The following data were extracted independently for each included article: first author, publication year, study population, burnout assessment, reported burnout, and main findings. *P*-values, confidence intervals, and odds ratios were extracted when available. Methodological quality of studies was assessed using the Newcastle-Ottawa Scale for cross-sectional studies [52], the Newcastle-Ottawa Scale for cohort studies [53], and the Cochrane Risk of Bias Tool for randomized controlled trials [54]. Study quality assessment is presented in Additional file 1: Tables S3, S4, S5, and S6.

### Findings

**Fig. 1:**
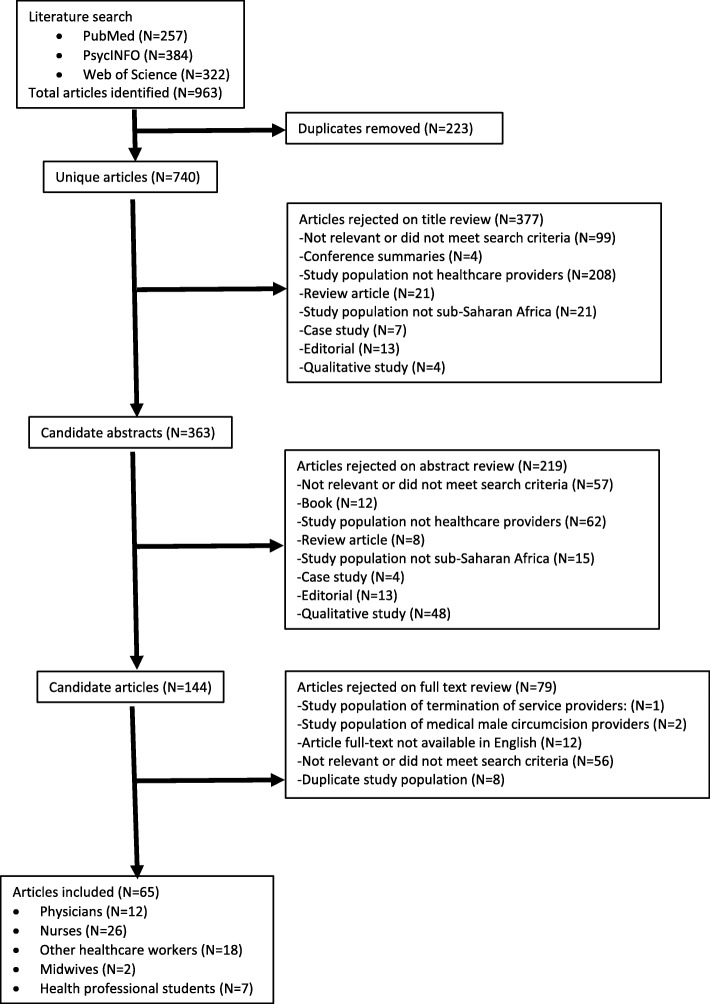
Flowchart of systematic literature review

The initial literature search identified a total of 233 unique articles in PubMed, 384 articles in PsycINFO, and 322 articles in the Web of Science database (Fig. 1). Duplicate articles were removed, and 740 unique articles remained for title review. Articles were rejected on title review if they were not relevant or did not meet search criteria. After reviewing article titles, 363 articles remained for abstract review. Candidate abstracts of the remaining studies were rejected for not being relevant or not meeting the search criteria. Studies in populations of healthcare providers (*N* = 144) were selected for full-text review. In the full-text review, articles were rejected if they were qualitative studies, not available in English, did not include healthcare providers, or were not relevant to the search criteria.

A total of 65 articles met our inclusion criteria for this systematic review. Included articles examined burnout in sub-Saharan Africa among physicians (*N* = 12 articles), nurses (*N* = 26), combined populations of healthcare workers (*N* = 18), midwives (*N* = 2), and medical or nursing students (*N* = 7). Twenty-seven studies examined burnout among healthcare providers in South Africa, 13 studies in Nigeria, 4 studies in Ethiopia, and 4 studies in Ghana. Three studies each examined burnout among healthcare providers in Cameroon and Malawi. Two studies each examined burnout among healthcare providers in Kenya and Zambia. One study each examined burnout in Zimbabwe, Botswana, Mozambique, Uganda, Namibia, and Senegal. Additionally, one study examined burnout in Kenya, Tanzania, and Uganda. Of the 65 eligible articles for inclusion, 45 used versions of the Maslach Burnout Inventory (MBI) to measure burnout. An additional 5 studies used the burnout subscale of the Professional Quality of Life Scale (ProQOL), 4 studies used the Oldenburg Burnout Inventory, 3 studies used the Copenhagen Burnout Inventory, 1 study used the Executive Burnout Scale, and 1 study used the Compassion Fatigue Self Test.

### Burnout among physicians

Twelve articles examined burnout among physicians in sub-Saharan Africa, comprising a total of 2031 participants across Ethiopia, Nigeria, Ghana, and South Africa (Table 1). In nine of the studies, burnout was assessed using the Maslach Burnout Inventory-Human Services Survey (MBI-HSS) [56, 57, 61, 66], MBI [55, 58, 60, 64], or an abbreviated MBI [59]. One study assessed burnout using the Copenhagen Burnout Inventory [63]. In South Africa, Schweitzer used one question (‘Do you ever feel so emotionally exhausted that you feel negative about yourself and about your job and lose the feeling of concern for your patients?’) to assess burnout based on the definition in Pine and Maslach [67]. Lastly in Nigeria, two questions were used to examine emotional exhaustion (‘I feel burned out from my work’) and depersonalization (‘I have become more callous toward people since I took this job’) [65].

**Table 1:** Characteristics of studies on burnout among physicians in sub-Saharan Africa (*N* = 12)

1st author, Year	Country	Study population	Burnout assessment	Reported burnout	Main findings
Coker, 2010 [55]	Nigeria	Physicians at a psychiatric hospital (*N* = 24)	MBI	12.5% reported burnout on emotional exhaustion, 33.3% on depersonalization, and 25% on low personal accomplishment. 23.6% reported high overall burnout.	8.3% of physicians also reported high scores on the Psycho-Physiological Symptoms Checklist.
Liebenberg, 2018 [56]	South Africa	Physicians at rural district hospitals (N = 36)	MBI-HSS	Emotional exhaustion (mean ± SD): 30.5 ± 11.0 Depersonalization: 14.6 ± 6.0 Personal accomplishment: 34.1 ± 6.0 81% reported burnout, with 31% reporting high burnout on all subscales.	Mean scores on the emotional exhaustion and depersonalization subscales were significantly greater than normative scores. Mean personal accomplishment scores did not differ from normative values.
Lrago, 2018 [57]	Ethiopia	Physicians at public hospitals (N = 491)	MBI-HSS	Emotional exhaustion (mean ± SD): 27.2 ± 8.0 Depersonalization: 12.9 ± 5.3 Personal accomplishment: 25.1 ± 6.6 65.2% reported high emotional exhaustion, 85.1% high depersonalization, and 91% low personal accomplishment.	Age, recognition from hospital managers, monthly salary, and number of patients observed per week were associated with emotional exhaustion (*p* < 0.05). Monthly salary and working in a primary hospital were associated with personal accomplishment (*p* < 0.05). Age, working in primary hospital, support from family and organization, monthly salary and professional training were associated with depersonalization (*p* < 0.05).
Ogundipe, 2014 [58]	Nigeria	Physicians undergoing residency training in a tertiary hospital (*N* = 204)	MBI	45.6% reported burnout on emotional exhaustion, 57.8% on depersonalization, 61.8% on personal accomplishment.	Participants who reported emotional distress were more likely to report burnout (OR = 6.97; 95% CI:3.28–14.81). Those who did not report doctor/doctor conflict were less likely to have depersonalization (OR = 0.36; 95% CI:0.17–0.76). Advanced age (OR = 0.66; 95% CI:0.47–0.95) and adequate support from management (OR = 0.45; 95% CI:0.22–0.90) were protective of burnout subscale of reduced personal accomplishment.
Opoku, 2014 [59]	Ghana	Physicians from web-based survey (*N* = 200)	Abbreviated MBI	Emotional exhaustion (mean ± SD): 9.1 ± 2.6 Depersonalization: 5.2 ± 2.1 Personal accomplishment: 5.8 ± 1.6 Total burnout: 20.0 ± 4.5	Overall career satisfaction (measured using physician work life survey) was negatively associated with emotional exhaustion (β = − 0.178, *p* < 0.001), low personal accomplishment (β = − 0.126, *p* < 0.01), and depersonalization (β = − 0.733, *p* < 0.05).
Peltzer, 2003 [60]	South Africa	Physicians (*N* = 402)	MBI	Emotional exhaustion (mean ± SD) 24.2 ± 10.8 Depersonalization: 11.4 ± 6.7 Personal accomplishment: 17.4 ± 6.8	The job stress index was found to be a predictor for emotional exhaustion (*p* < 0.001) and depersonalization (*p* < 0.001) but not personal accomplishment. Sex, age, race, length of service, and marital status were significantly associated with burnout subscales (*p* < 0.05).
Rajan, 2018 [61]	South Africa	Physicians working in public sector emergency centers (*N* = 93)	MBI-HSS	Emotional exhaustion (mean ± SD): 31.7 ± 10.3 Depersonalization: 13.4 ± 6.2 Personal accomplishment: 34.9 ± 6.5	Sex and relationship status were not significantly associated with burnout scores. There were significantly higher depersonalization scores among physicians in the moderate to high risk group who were less than 40 years of age, compared to those who were 40 years old and above (87% vs 61%, *p* < 0.05). Those with two or less years of experience had a significantly higher probability of leaving in the next five years compared to those with more experience (62% vs. 39%, *p* < 0.05).
Schweitzer, 1994 [62]	South Africa	Junior physicians (*N* = 126)	One question worded: “Do you ever feel so emotionally exhausted that you feel negative about yourself and about your job and lose the feeling of concern for your patients?”	77.8% had experienced burnout, 52.4% were experiencing burnout at current job, and 61% experienced burnout at a previous job.	Physician Stress Inventory (PSI) score was significantly higher among participants with burnout (*p* < 0.001). Doctors who were able to communicate with the majority of patients had lower burnout than those who could not (*p* = 0.04) and a lower mean PSI score (*p* = 0.04).
Stassen, 2013 [63]	South Africa	Advanced life support paramedics (*N* = 40)	CBI	Work related burnout (mean ± SD): 44.3 ± 16.8 Personal burnout: 48.0 ± 16.7 Patient care related burnout: 35.6 ± 16.2 Overall burnout: 42.9 ± 14.0 38% reported work related burnout, 53% reported personal burnout, 23% reported patient care related burnout, and 30% reported overall burnout .	Burnout was not significantly associated with gender, employment sector, years of experience, or qualifications.
Stodel, 2011 [64]	South Africa	Junior physicians at a children’s hospital (*N* = 22)	MBI	Emotional exhaustion (mean ± SD): 37.7 ± 8.9 Depersonalization: 12.6 ± 5.6 Personal accomplishment: 32.1 ± 5.8	The mean scores on the emotional exhaustion (*p* = 3.29 × 10^− 13^) and depersonalization (*p* = 2.35 × 10^− 7^) subscales were significantly higher compared to a normative sample. Among surveyed participants, 95% reported an intention to leave the hospital.
Ugwu, 2019 [65]	Nigeria	Physicians at intensive care units of hospitals (*N* = 183)	Items that had the highest factor loading on emotional exhaustion (‘I feel burned out from my work’) and depersonalization (‘I have become more callous toward people since I took this job’)	5.5 ± 1.9 (mean ± SD)	Job burnout was significantly related to recovery from job stressors (*p* < 0.001), and perceived family cohesion (*p* < 0.01).
van der Walt, 2015 [66]	South Africa	Anesthetists at a university hospital (*N* = 124) and in private practice (*N* = 86)	MBI-HSS	Among hospital anesthetists, 45.2% reported high emotional exhaustion, 50% reported high depersonalization, and 46% reported low personal accomplishment. Among private practice anesthetists, 20.9% reported high emotional exhaustion, 26.7% reported high depersonalization, and 37.2% reported low personal accomplishment. High burnout was identified in 21% of hospital anesthetists and 8.1% of anesthetists in private practice.	Among anesthetists, burnout was not significantly associated with age, gender, or years of experience.

*Abbreviations*: *CBI* Copenhagen Burnout Inventory, *MBI* Maslach Burnout Inventory, *MBI-HSS* Maslach Burnout Inventory - Human Services Survey

Physicians reported high levels of burnout. For example, among physicians at rural district hospitals in South Africa (*N* = 36), 81% of participants reported burnout, with 31% reporting high burnout on all three of the MBI-HSS subscales [56]. On the MBI-HSS subscales, 65.2% of physicians in southern Ethiopia (*N* = 491) reported high emotional exhaustion, 91% low personal accomplishment, and 85.1% high depersonalization [57]. Physicians undergoing residency training at a hospital in Nigeria (*N* = 204) reported a high prevalence of burnout according to the MBI, in which 45.6% of residents reporting burnout on emotional exhaustion, 57.8% depersonalization, and 61.8% reduced personal accomplishment [58]. Among physicians who participated in the web-based survey in Ghana (*N* = 200), burnout measures were high on the emotional exhaustion (mean ± standard deviation (SD): 9.1 ± 2.6), personal accomplishment (5.8 ± 1.6), and depersonalization (5.2 ± 2.1) subscales of the abbreviated MBI [59]. South African physicians in public sector emergency centers (*N* = 93) had high burnout scores on all subscales of the MBI-HSS [61]. Among physician anesthetists at a university hospital in South Africa, 45.2% reported high emotional exhaustion, 50% reported high depersonalization, and 46% reported low personal accomplishment on the MBI-HSS [66]. Among South African anesthetists in private practice, 20.9% reported high emotional exhaustion, 26.7% reported high depersonalization, and 37.2% reported low personal accomplishment on the MBI-HSS [66]. In a population of junior physicians in South Africa (*N* = 126), 77.8% had experienced burnout, with 52.4% experiencing burnout at their current job. Among these doctors, scores on the Physician Stress Inventory were significantly higher among those with burnout (*p* < 0.001) [62]. Lastly, in a small mixed-methods study of junior physicians at a children’s hospital in South Africa (*N* = 22), all participants experienced high levels of burnout on at least 1 MBI subscale, and mean scores on the emotional exhaustion and depersonalization subscales were significantly higher than those in a normative comparison group (*p* < 0.001) [64].

### Burnout among nurses

A total of 26 articles examined burnout among nurses in Ghana, South Africa, Nigeria, Kenya, Tanzania, Uganda, Cameroon, Namibia, and Zimbabwe (Table 2). The majority of studies were conducted in South African (*N* = 13) or Nigerian (*N* = 8) nursing populations. Of the 26 articles, a total of 20 studies used the Maslach Burnout Inventory-General Survey (MBI-GS), MBI-HSS, MBI, or the MBI emotional exhaustion subscale to measure burnout. Two studies used the Oldenburg Burnout Inventory [74, 85], one study used the burnout subscale of the ProQOL [83], and one study used first-hand coding by an observer according to the Exhaustion-Disengagement Model [84], which uses job demand and resources to identify exhaustion and disengagement. One study used the Executive Burnout Scale, which was developed in Nigeria as a culturally-sensitive tool to measure burnout [68, 95]. One study did not specify the burnout measure used [73]. A total of 5 studies did not report measured burnout levels in the study population [79, 84, 86, 89, 94].

**Table 2:** Characteristics of studies on burnout among nurses in sub-Saharan Africa (*N* = 26)

1st author, Year	Country	Study population	Burnout assessment	Reported burnout	Main findings
Amoo, 2008 [68]	Nigeria	Psychiatric nurses (*N* = 50) and secondary school teachers (*N* = 50)	Executive Burnout Scale	Among nurses, Total burnout (mean ± SD): 47.4 ± 12.2 General subscale: 21.3 ± 5.7 Somatic subscale: 16.4 ± 5.9 Interpersonal subscale: 9.9 ± 3.0	Teachers had significantly higher total job burnout, and burnout on the three subscales (general, somatic, and interpersonal) than nurses (*p* < 0.05). Burnout was not associated with sex, marital status, age and length of service. No significant difference in job satisfaction was observed between the two groups (*p* = 0.297).
Asiedu, 2018 [69]	Ghana	Nurses from public hospitals (*N* = 134)	MBI-GS	1.7 ± 0.8 (mean ± SD)	Sex, age, number of older dependents, weekend work, work-to-family conflict and family-to-work conflict were significantly associated with burnout (*p* < 0.05). Work-to-family conflict and family-to-work conflict accounted for 20% of variance in burnout.
Buitendach, 2011 [70]	Namibia	Nurses from two private hospitals (*N* = 191)	MBI-GS	Exhaustion (mean ± SD): 11.3 ± 8.6 Cynicism: 4.6 ± 4.8 Professional efficacy: 25.5 ± 10.5	Job satisfaction was associated with emotional exhaustion and cynicism. The interaction of problem-focused coping and job satisfaction were significant predictors of emotional exhaustion (*p* < 0.05)
Coetzee, 2013 [71]	South Africa	Nurses at private and public national referral hospitals (*N* = 1187)	Emotional Exhaustion subscale of MBI	45.8% report high levels of burnout on emotional exhaustion subscale	Nurses with more favorable practice environments were less likely to report high burnout (OR = 0.55; 95% CI: 0.41–0.75). Nurses who worked at public hospitals were more likely to have burnout compared to those at private hospitals (53.8% vs. 40.6%; *p* < 0.001).
Davhana-Maselesele, 2008 [72]	South Africa	Nurses caring for HIV-positive and AIDS patients (*N* = 174)	MBI	Mean for personal accomplishment, emotional exhaustion and depersonalization were 52, 33 and 29%, respectively	High measures of depression, sadness, fatigue and low energy were found among nurses.
Engelbrecht, 2008 [73]	South Africa	Nurses at clinics and community health centers (*N* = 542)	MBI-HSS	Emotional exhaustion (mean ± SD): 31.3 ± 9.3 Depersonalization: 17.8 ± 4.9 Personal accomplishment: 20.3 ± 6.8	Availability of resources, time pressure of workload, and conflict and social relations predicted 21% of the variance in emotional exhaustion and 8% of the variance in depersonalization scores. Availability of resources and time pressure of workload predicted 14% of variance in personal accomplishment.
Ezenwaji, 2019 [74]	Nigeria	Nurses at hospitals (*N* = 393)	Oldenburg Burnout Inventory	Mean burnout score of male nurses was 3.2 ± 0.1 and female nurses was 3.2 ± 0.1	Sex, age, work experience, and work environment were not significantly associated with burnout scores.
Gandi, 2011 [75]	Nigeria	Nurses at hospitals (*N* = 373)	MBI-GS	Among men: Emotional exhaustion (mean ± SD):2.3 ± 1.3 Depersonalization: 0.6 ± 0.7 Personal accomplishment: 5.1 ± 1.1 Among women: Emotional exhaustion (mean ± SD): 2.5 ± 1.3 Depersonalization: 0.8 ± 0.9 Personal accomplishment: 5.2 ± 0.8	Sex was not significantly associated with burnout scores. The relationship between work characteristics and burnout was mediated by work-home interference and home-work interference.
Gorgens-Ekermans, 2012 [76]	South Africa	Nurses (*N* = 122)	MBI	Emotional exhaustion (mean ± SD): 13.6 ± 11.0 Depersonalization: 6.6 ± 5.3 Personal accomplishment: 34.1 ± 9.9	Emotional management and emotional control, as measured by the Swinburne University Emotional Intelligence test, were associated with self-reported stress and burnout subscales (*p* < 0.01). Workload was a significant predictor of emotional exhaustion (β = 0.547, *p* = < 0.001) and work/family interface as a source of stress was a significant predictor of depersonalization (β = 0.296, *p* = 0.004). Emotional intelligence was a moderator of the relationship between stress and burnout, explaining 59.5% of the variance in the emotional exhaustion and 23.9% of the variance in the depersonalization subscale of burnout.
Heyns, 2003 [77]	South Africa	Nurses caring for patients with Alzheimer’s disease (*N* = 226)	MBI	Emotional exhaustion (mean ± SD): 14.3 ± 10.3 Depersonalization: 4.5 ± 5.6 Personal accomplishment: 36.3 ± 8.2 26% reported high emotional exhaustion, 21% high depersonalization, and 66% low personal accomplishment.	Sense of Coherence Scale, Fortitude Questionnaire scores, age, years of experience, hours of work, hours of direct attention to patients, qualifications and institution predicted scores on the burnout subscales (*p* < 0.01).
Ifeagwazi, 2005 [78]	Nigeria	Nurses from a teaching hospital (*N* = 91)	MBI	Total burnout (mean ± SD): widowed nurses: 3.1 ± 0.3 married nurses: 2.6 ± 0.5	Widowed nurses reported significantly higher burnout than married nurses (*p* < 0.001). There were significant differences between hospital units on mean burnout symptoms reported (*p* < 0.01), with nurses on the operating theater unit having higher mean burnout scores than nurses on the postnatal, casualty, labor, surgical and out-patient units. Nurses on intensive care unit had higher mean burnout than on the postnatal unit.
Khamisa, 2015 [79]	South Africa	Nurses from two private and two public hospitals (*N* = 895)	MBI-HSS	Not reported	Staffing issues explain the highest variance in emotional exhaustion (16%), depersonalization (13%) and personal accomplishment (10%) subscales. Emotional exhaustion and personal accomplishment are associated with somatic symptoms explaining 21% of the variance in general health. In a follow-up survey, lack of support is associated with burnout (OR = 4.37, 95% CI: 2.89–6.62), and patient care is associated with job satisfaction (OR = 2.63, 95% CI: 1.35–5.16) [84].
Lasebikan, 2012 [81]	Nigeria	Hospital nurses (*N* = 270)	MBI	39.1% had high burnout on the emotional exhaustion subscale, 29.2% in depersonalization and 40.0% on reduced personal accomplishment.	Doctor/nurse conflict (OR = 3.1, 95% CI: 1.9–6.3), inadequate nursing personnel (OR = 2.6, 95% CI: 1.5–5.1), and frequent night duties (OR = 3.1, 95% CI: 1.7–5.6) were predictors of burnout on the emotional exhaustion subscale. Doctor/nurse conflict (OR = 3.4, 95% CI: 2.2–7.6) and frequent night duties (OR = 2.4, 95% CI: 1.5–4.8) were predictors of burnout on the depersonalization subscale. High nursing hierarchy (OR = 2.7, 95% CI: 1.5–4.8), poor wages (OR = 2.9, 95% CI: 1.6–5.6), and frequent night duties (OR = 2.3, 95% CI: 2.3–4.5) were predictors of burnout on the reduced personal accomplishment subscale.
Levert, 2000 [82]	South Africa	Nurses at psychiatric hospitals (*N* = 94)	MBI	Emotional exhaustion (mean ± SD): 29.9 ± 12.9 Depersonalization: 9.6 ± 4.6 Personal accomplishment: 19.2 ± 8.3	Emotional exhaustion was associated with nurses’ workload, lack of support from colleagues, role conflict and role ambiguity (*p* < 0.05). Personal accomplishment was associated with role conflict (*p* = 0.015). Depersonalization was associated with work load, lack of support from colleagues, role conflict and role ambiguity (*p* < 0.05).
Mashego, 2016 [83]	South Africa	Hospital nurses (*N* = 83)	ProQOL, burnout subscale	30.7 ± 5.3 (mean ± SD)	92% had moderate burnout. Burnout score was not associated with age, marital status, education level, or years of working in the maternity ward.
Mbambo, 2003 [84]	South Africa	Nurses in a District Health System (*N* = 60)	Observer coded according to Exhaustion-Disengagement Model	Not reported	Hospital nurses have higher job demands and lower job resources compared to primary healthcare nurses. Hospital nurses run a greater risk of exhaustion and disengagement.
Mbanga, 2018 [85]	Cameroon	Nurses at state-owned and private hospitals (*N* = 143)	Oldenburg Burnout Inventory	38.4 ± 5.7 (mean ± SD)	In univariable regression analyses, being in a relationship was significantly protective, predicting 3.8% of variation in burnout syndrome (*p* = 0.029).
Mefoh, 2019 [86]	Nigeria	Nurses at a tertiary healthcare hospital (*N* = 283)	MBI-HSS	Not reported	Emotion-focused coping was positively associated with burnout subscales of emotional exhaustion (β = 0.32, *p* = 0.01), and depersonalization (β = 0.18, *p* = 0.01). Emotion focused coping was not significantly associated with burnout subscale of reduced personal accomplishment (β = − 0.10, *p* = 0.45). However, the interaction effect of age and emotion-focused coping on reduced personal accomplishment was significant (β = 0.03, *p* = 0.04).
Okwaraji, 2014 [87]	Nigeria	Nurses at a tertiary health institution (*N* = 210)	MBI	42.9% high emotional exhaustion, 47.6% depersonalization, and 53.8% reduced personal accomplishment.	Burnout was significantly higher among nurses who were women, older than 35 years old, not married, and those with nursing certificates compared to those with nursing degrees or nursing officers (*p* < 0.01).
Pienaar, 2011 [88]	South Africa	Nurses from 225 clinics (*N* = 542)	MBI	Emotional exhaustion (mean ± SD): 31.3 ± 9.3 Depersonalization: 17.8 ± 4.9 Personal accomplishment: 20.3 ± 6.8	Burnout subscale scores were associated with intention to quit nursing jobs (*p* < 0.001)
Roomaney, 2017 [89]	South Africa	Nurses at a large tertiary hospital (*N* = 110)	MBI	Not reported	Workload, job status, and interpersonal conflict at work significantly explained more than one-third of the variance on the emotional exhaustion subscale of burnout (R^2^ = 0.39, *p* = 0.001). Interpersonal conflict, workload, organizational constraints and HIV stigma significantly explained the depersonalization subscale (R^2^ = 0.33, *p* = 0.001). Job status and organizational constraints significantly predicted personal accomplishment subscale (R^2^ = 0.18, *p* = 0.001).
van der Colff, 2014 [90]	South Africa	Nurses in private, public, hospital, community, psychiatric and management sectors (*N* = 818)	MBI	Emotional exhaustion (mean ± SD): 22.2 ± 11.3 Depersonalization: 7.2 ± 5.9 Personal accomplishment: 34.5 ± 7.6	Exploratory factor analysis resulted in a three-factor structure of burnout. Statistically significant differences were found in burnout levels with regard to language, age, rank, job satisfaction, reciprocity, full-time employment and specialized training (*p* < 0.01).
van der Doef, 2012 [91]	Kenya, Tanzania, and Uganda	Nurses in private and public hospitals (N = 309)	MBI	32.1% reported burnout	In comparison with a reference Dutch population, the East African nurses have higher emotional exhaustion (t = 13.2, *p* < 0.001) and depersonalization (t = 3.60, *p* < 0.001). East African nurses had lower scores on personal accomplishment than the reference population (t = 11.34, *p* < 0.001). Job conditions explain 17% of the variance on the emotional exhaustion subscale. A higher workload (β = −0.21, *p* < 0.01), lower social support from colleagues (β = − 0.15, *p* < 0.05) and problems concerning information provision (β = − 0.20, *p* < 0.001) are associated with higher emotional exhaustion. 7.4% of the variance in personal accomplishment is explained by job conditions. Higher decision latitude (β = − 0.15, *p* < 0.05) and better interdepartmental cooperation (β = − 0.17, *p* < 0.05) are associated with higher personal accomplishment. Job conditions fail to explain a significant proportion of the variance on depersonalization.
van Doorn, 2016 [92]	Nigeria	Nurses at an international health organization (*N* = 214)	Emotional exhaustion subscale of the MBI	4.8 ± 1.6 (mean ± SD)	Emotional exhaustion was significantly associated with gender, age, job demands, and lack of supervisor support (*p* < 0.01).
van Wijk, 1997 [93]	South Africa	Nurses at military institutions (*N* = 46)	Not specified	34% reported a ‘burnout experience’ within the past 3 months	Burnout was more common among registered nurses (46%) compared to enrolled (35%) or assistant nurses (21.4%). Nurses in isolated areas had higher burnout compared to nurses in more populated areas (44 vs. 26%, respectively). Burnout was higher among younger nurses.
Wilson, 1989 [94]	Zimbabwe	Nurses (*N* = 83)	MBI	Not reported	Internal-External externality score was significantly related to personal accomplishment subscale (r = −0.24, *p* < 0.05), depersonalization subscale (r = 0.03, *p* < 0.05), and total burnout (r = 0.20, *p* < 0.05) but unrelated to the emotional exhaustion subscale (r = 0.03).

*Abbreviations*: *MBI* Maslach Burnout Inventory, *MBI-HSS* Maslach Burnout Inventory - Human Services Survey, *MBI-GS* Maslach Burnout Inventory – General Survey

High levels of reported burnout were found in nursing populations (Table 2). For example, in a large study of nurses at national referral hospitals in South Africa (*N* = 1187), 45.8% participants reported high levels of burnout on the emotional exhaustion subscale of the MBI [71]. Among hospital nurses in Nigeria (*N* = 270), 39.1% had burnout on the emotional exhaustion subscale of the MBI, 29.2% on the depersonalization subscale, and 40.0% on the reduced personal accomplishment subscale [81]. In a population of nurses at private and public hospitals in Kenya, Tanzania, and Uganda, (*N* = 309), 32.1% reported burnout on the MBI [91]. Among nursing populations in South Africa, burnout was associated with high workloads [73, 76, 82, 89] and lack of support [79, 80, 91, 92].

### Burnout among combined populations of healthcare workers

A total of 18 articles examined burnout among combined populations of healthcare workers (Table 3). Three studies each were conducted in Ethiopia and Malawi. Two studies each were conducted in Nigeria, Ghana, Zambia, South Africa, and Kenya. One study each was conducted in Botswana and Mozambique. A total of 12 studies used the MBI, MBI-GS, or MBI-HSS to assess burnout [96, 99–101, 104–106, 108–112]. For example, in a small sample of healthcare workers in a trauma unit in South Africa (*N* = 38), 61% had high emotional exhaustion, 50% high depersonalization, and 50% reduced personal accomplishment on MBI subscales [99]. Among healthcare workers providing clinical care for HIV-positive patients in Malawi (*N* = 520), 62% met the MBI criteria for burnout [101]. Additionally, one study used the Compassion Fatigue Self Test [102] and one used the Copenhagen Burnout Inventory [97]. Two studies measured burnout as a sub-domain of motivation [98, 113]. One study measured burnout using a five item scale of occupational burnout [103]. Mutale and colleagues used two questions to measure burnout (“I feel emotionally drained at the end of the day” and “Sometimes when I get up in the morning, I dread having to face another day at work”) [107]. In combined populations of healthcare workers, nurses often had the highest level of reported burnout [97, 111].

**Table 3:** Characteristics of studies on burnout among combined populations of healthcare workers in sub-Saharan Africa (*N* = 18)

First Author, Year	Country	Study population	Burnout assessment	Reported burnout	Main findings
Bhagavathula, 2018 [96]	Ethiopia	Healthcare workers at a teaching hospital (*N* = 248)	MBI	Emotional exhaustion (mean ± SD): 5.4 ± 1.2 Inefficacy: 5.1 ± 1.7 Cynicism: 4.8 ± 2.0 13.7% reported overall burnout.	Burnout was associated with age (*p* = 0.008), number of patients treated per day (*p* < 0.001), and shift work (*p* < 0.001). In multivariable analyses, sex, marital status, profession, and work experience were significantly associated with burnout subscales (*p* < 0.01).
Biksegn, 2016 [97]	Ethiopia	Healthcare workers at a teaching hospital (*N* = 334)	CBI	50.3 ± 17.2 (mean ± SD)	Nurses had the highest prevalence (82.8%) of burnout and laboratory technicians had the lowest (2.8%). Job insecurity, history of physical illness, low interest in profession, poor relationship status with managers, worry of contracting infection or illness and physical/verbal abuse were predictors of burnout.
Bonenberger, 2014 [98]	Ghana	Healthcare workers (*N* = 256)	Instrument to measure motivation with 7 outcome constructs, including burnout	3.3 ± 1.0 (mean ± SD)	Motivation and job satisfaction were significantly associated with career development (OR = 0.56, 95% CI: 0.36–0.86), workload (OR = 0.58, 95% CI: 0.34–0.99), management (OR = 0.51, 95% CI: 0.30–0.84), organizational commitment (OR = 0.36, 95% CI: 0.19–0.66), and burnout (OR = 0.59, 95% CI: 0.39–0.91).
Crabbe, 2004 [99]	South Africa	Healthcare workers in trauma unit of a hospital (*N* = 38)	MBI	61% had high emotional exhaustion, 50% high depersonalization, and 50% high reduced personal accomplishment	At least half of respondents reported high professional burnout in all 3 MBI subscales.
Fiadzo, 1997 [100]	Ghana	Healthcare workers (*N* = 287)	MBI	Not reported	Study provides support for burnout progression model
Kim, 2018 [101]	Malawi	Healthcare workers providing clinical care for HIV-positive patients (*N* = 520)	MBI	62% met criteria for total burnout, with 55% reporting moderate-high emotional exhaustion, 31% moderate-high depersonalization, and 46% low-moderate personal accomplishment.	Burnout was associated with self-reported suboptimal patient care (OR = 3.22, 95% CI: 2.11–4.90; *p* < 0.0001)
Kokonya, 2014 [102]	Kenya	Healthcare workers at a national hospital (*N* = 345)	Compassion Fatigue Self-Test	95.4% reported high burnout	96.7% of medical practitioners and 94.1% of nurses reported high burnout. Burnout was not significantly associated with participants’ sex, age, marital status, religion, education, or number of years as a healthcare provider.
Kruse, 2009 [103]	Zambia	Healthcare providers (*N* = 483 active clinical staff completed questionnaire; *N* = 50 in focus groups, *N* = 4 interviews)	Occupational burnout measured on 5-item scale	51% of respondents reported occupational burnout	Occupational burnout was associated with having another job (RR = 1.4, 95% CI: 1.2–1.6) and knowing a co-worker who left in the last year (RR = 1.6, 95% CI: 1.3–2.2).
Ledikwe, 2018 [104]	Botswana	Healthcare workers at a public health facility (*N* = 1348)	MBI-GS	Professional efficacy (mean ± SD): 4.9 ± 1.1 Exhaustion: 2.3 ± 1.7 Cynicism: 2.4 ± 1.4	Overall job satisfaction assessed by the Job In General Scale was significantly higher for healthcare workers who participated in 7 or more activities as part of the Botswana’s Workplace Wellness Program (WWP) compared with those who did not participate in any activities (*p* = 0.004). Stress levels (*p* = 0.006), measured on the Stress in General scale, and exhaustion (*p* < 0.001), measured on the MBI, were significantly lower among those with high participation in WWP activities.
Madede, 2017 [105]	Mozambique	Healthcare workers (quantitative: *N* = 92 baseline and 49 post-intervention; *N* = 17 qualitative interviews)	MBI	At baseline, 67.1% low, 15.9% moderate, and 17.1% high burnout. After intervention, 71.1% low, 17.8% moderate, 11.1% high burnout.	There were no significant differences in emotional exhaustion between baseline and post intervention, for any intervention groups. Job satisfaction, emotional exhaustion and work engagement showed no significant differences between baseline and post intervention.
McAuliffe, 2009 [106]	Malawi	Healthcare workers in public and private facilities (*N* = 153)	MBI	31% reported high emotional exhaustion, 5% reported high depersonalization, and 45% reported low personal accomplishment	The adequate resources subscale of the Health Care Providers Work Index correlates with emotional exhaustion on the MBI.
Mutale, 2013 [107]	Zambia	Healthcare workers from health facilities (*N* = 96)	“I feel emotionally drained at the end of the day” and “Sometimes when I get up in the morning, I dread having to face another day at work.”	Not reported	Burnout was higher among women as compared to men in 2 of the 3 districts. Linear regressions showed major determinants of higher motivation were female (*p* = 0.008) and working in non-clinical areas (for example, pharmacists or laboratory technicians, *p* = 0.039).
Ndetei, 2008 [108]	Kenya	Healthcare workers at a psychiatric hospital (*N* = 121)	MBI-HSS and MBI-GS	Emotional exhaustion (mean ± SD): 17.2 ± 9.8 Depersonalization: 7.3 ± 5.8 Personal accomplishment: 29.3 ± 10.3	Emotional exhaustion was significantly associated with younger age (*p* < 0.001), number of children (*p* = 0.003), number of years worked (*p* = 0.049), heavy workload (*p* < 0.001) and low morale (*p* = 0.001). Depersonalization was significantly associated with heavy workload (*p* = 0.034). Reduced personal accomplishment was associated with younger age (*p* = 0.03).
Nel, 2013 [109]	South Africa	Healthcare workers at public and private hospitals (*N* = 511)	MBI-HSS	Emotional exhaustion (mean ± SD): 15.2 ± 7.2 Mental distance: 13.6 ± 9.3	The proposed structural model shows paths between job demands and job resources; job demands, emotional intelligence and work wellness; job resources, emotional intelligence and work wellness.
Ojedokun, 2013 [110]	Nigeria	Healthcare workers working in AIDs care (*N* = 242)	MBI	66.4 ± 21.5 (mean ± SD)	Burnout was significantly associated with aggressive tendency and perceived fear of AIDS (*p* < 0.01)
Olley, 2003 [111]	Nigeria	Healthcare workers at a teaching hospital (*N* = 260)	MBI	Not reported	Nurses reported higher scores on burnout subscales compared to other healthcare providers (*p* < 0.05). Significant differences were found between nurses and other healthcare providers on the General Health Questionnaire-12 (*p* < 0.01) and the State Trait Anxiety Inventory (*p* < 0.05).
Thorsen, 2011 [112]	Malawi	Healthcare workers in a referral hospital (*N* = 101)	MBI-HSS	Emotional exhaustion (mean ± SD): 23.1 ± 9.7 Depersonalization: 6.2 ± 4.8 Personal accomplishment: 37.8 ± 7.5	Sociodemographic characteristics were not associated with the emotional exhaustion subscale of burnout. For the depersonalization and personal accomplishment subscales, number of children was the only significant predictor (*p* < 0.05).
Weldegebriel, 2016 [113]	Ethiopia	Healthcare workers at public hospitals (*N* = 304)	Organizational burnout measured as a subdimension of motivation	3.6 ± 1.3 (mean ± SD)	Performance review was the only significant predictor of the burnout dimension of motivation. Respondents who never had a performance review conducted had an average decrease of 0.155 units (95% CI: −0.875 to −0.122) in burnout motivation score as compared to those with formal performance assessment.

Abbreviations: *MBI* Maslach Burnout Inventory, *MBI-HSS* Maslach Burnout Inventory - Human Services Survey, *MBI-GS* Maslach Burnout Inventory - General Survey

### Burnout among midwives

Two studies examined burnout among midwives in Uganda [114] and Senegal [115] (Table 4). Among midwives in two rural districts in Uganda (*N* = 224), burnout was measured using the burnout subscale of the Professional Quality of Life Scale [114]. Burnout and secondary traumatic stress were associated with level of education (*p* < 0.01), marital status (*p* < 0.01), involvement in non-midwifery health care activities (*p* < 0.01), and physical well-being (*p* < 0.01) [114]. Among midwives from 22 hospitals in Senegal (*N* = 226), 55% reported burnout on the MBI, with 80% reporting burnout on emotional exhaustion, 57.8% on depersonalization, and 12.4% on diminished personal accomplishment subscales. Furthermore, emotional exhaustion was inversely associated with remuneration (*p* = 0.02) and task satisfaction (*p* = 0.03). Active job searching was associated with being dissatisfied with job security (*p* < 0.01), and voluntary quitting was associated with dissatisfaction with continuing education (*p* < 0.01) [115].

**Table 4:** Characteristics of studies on burnout among midwives and health professional students in sub-Saharan Africa (*N* = 9)

First Author, Year	Country	Study population	Burnout assessment	Reported burnout	Main findings
Midwives (*N* = 2)					
Muliira, 2016 [114]	Uganda	Midwives in two rural districts (*N* = 224)	ProQOL, burnout subscale	36.9 ± 6.2 (mean ± SD)	Compassion satisfaction was associated with psychological well-being (*p* < 0.01) and job satisfaction (*p* < 0.01). Burnout and secondary traumatic stress were associated with education level (*p* < 0.01), marital status (*p* < 0.01), involvement in non-midwifery healthcare (*p* < 0.01), and physical well-being (*p* < 0.01).
Rouleau, 2012 [115]	Senegal	Midwives from 22 hospitals (*N* = 185)	MBI	Emotional exhaustion (mean ± SD): 35.4 ± 9.6 Depersonalization: 11.4 ± 6.1 Personal accomplishment: 39.7 ± 4.8	Emotional exhaustion was inversely associated with remuneration (*p* = 0.02) and task satisfaction (*p* = 0.03). Actively job searching was associated with being dissatisfied with job security (*p* < 0.01), and voluntary quitting was associated with dissatisfaction with continuing education (*p* < 0.01).
Medical and nursing students (*N* = 7)					
Colby, 2018 [116]	South Africa	Medical students (*N* = 91)	MBI-HSS	41.7% had moderate burnout on the depersonalization subscale. 58.2% had high burnout on the personal accomplishment. Equal numbers of participants reported low or high emotional exhaustion (39.6 and 39.6%, respectively). Overall, 46.1% reported high, 33.8% moderate, and 20% low burnout.	There were significant associations between the psychological health subscale of the World Health Organization Quality of Life Assessment and all subscales of the MBI, in particular emotional exhaustion (*p* < 0.01).
Gordon, 2016 [117]	South Africa	Oral hygiene students (*N* = 89)	MBI	Emotional exhaustion (mean ± SD): 3.3 ± 1.8 Depersonalization: 1.3 ± 1.6 Personal accomplishment: 3.7 ± 1.7	There were significant differences in burnout between 1st, 2nd, and 3rd year students (*p* = 0.039).
Mason, 2012 [118]	South Africa	Nursing students (*N* = 80)	ProQOL, burnout subscale	63.75% had a moderate to high risk for burnout	Burnout was significantly associated with compassion fatigue and negatively associated with compassion satisfaction (*p* < 0.01).
Mathias, 2017 [119]	South Africa	Undergraduate nursing students (*N* = 67)	ProQOL, burnout subscale	6% had low levels of burnout, 94% moderate, & none had high burnout	The majority of nursing students experienced average levels of burnout, compassion fatigue, and compassion satisfaction.
Njim, 2018 [120]	Cameroon	Nursing students (*N* = 447)	Oldenburg Burnout Inventory	Disengagement (mean ± SD): 17.1 ± 3.1 Exhaustion: 20.9 ± 3.0	Satisfaction with results and regret with choice of nursing studies were determinants of burnout (*p* < 0.05)
Njim, 2019 [121]	Cameroon	Medical students (*N* = 413)	Oldenburg Burnout Inventory	Disengagement (mean ± SD): 16.6 ± 3.4 Exhaustion: 20.5 ± 3.5	Marital status, relationship difficulties, cumulative GPA, regretting the choice of medical studies, and recreational drug use significantly predicted burnout (*p* < 0.05).
Stein, 2016 [122]	South Africa	Paramedic students (*N* = 93)	CBI	Work related burnout (mean ± SD): 49.1 ± 12.9 Personal burnout: 53.4 ± 15.0 Patient care related burnout: 34.0 ± 19.5 Overall burnout: 45.2 ± 11.5 31% reported high burnout	There were no significant differences in mean burnout between the 4 academic years of study in work-related, personal, and patient care-related burnout.

*Abbreviations*: *CBI* Copenhagen Burnout Inventory, *MBI* Maslach Burnout Inventory, *MBI-HSS* Maslach Burnout Inventory - Human Services Survey, *ProQOL* Professional Quality of Life Scale

### Burnout among health professional students

Lastly, 7 articles examined burnout among medical and nursing students in South Africa or Cameroon (Table 4). Among medical students in South Africa (*N* = 91), 46.1% reported high, 33.8% moderate, and 20% low burnout on the MBI-HSS burnout scale [116]. Colby and co-authors also found significant associations between scores on the World Health Organization Quality of Life Assessment and the MBI subscales (p < 0.01) [116]. Among oral hygiene students in South Africa, there were significant differences in burnout levels on the MBI subscales between 1st, 2nd, and 3rd year students (*p* = 0.039) [117]. Among nursing students in South Africa (*N* = 80), 63.8% had a moderate to high risk of burnout [118]. In a population of undergraduate nursing students in South Africa (*N* = 67), Mathias and coauthors found on the burnout subscale of the Professional Quality of Life Scale (ProQOL) that 6% of participants had low levels of burnout, 94% had moderate, and none reported high levels of burnout [119]. Among nursing students (*N* = 447) and medical students (*N* = 413) in Cameroon, burnout was examined using the Oldenburg Burnout Inventory [120, 121]. Lastly, in a population of paramedic students in South Africa (*N* = 93), 31% of participants reported high levels of burnout on the Copenhagen Burnout Inventory [122].

### Risk and protective factors associated with burnout among healthcare providers

Overall, burnout was associated with measures of the work environment, including heavy workload, inadequate personnel, difficult work conditions, and low career satisfaction. For example, nurses in South Africa with more favorable work environments were less likely to report high levels of burnout (OR = 0.55; 95% CI: 0.41–0.75) [71]. Heavy workloads were also significantly associated with high levels of reported burnout in populations of nurses [76, 82, 89, 91, 123] and other healthcare workers [108, 109]. Among nurses in South Africa, workload was a significant predictor of emotional exhaustion as measured by the MBI (β = 0.547,*p* = < 0.001) [76]. Among hospital workers in Nigeria, inadequate number of nursing personnel (OR = 2.6, 95% CI: 1.5–5.1), and frequent night duties (OR = 3.1, 95% CI: 1.7–5.6) were predictors of burnout on the emotional exhaustion subscale of the MBI. Frequent night duties (OR = 2.4, 95% CI: 1.5–4.8) were predictors of burnout on the depersonalization subscale. High nursing hierarchy (OR = 2.7, 95% CI: 1.5–4.8), poor wages (OR = 2.9, 95% CI: 1.6–5.6), and frequent night duties (OR = 2.3, 95% CI: 2.3–4.5) were predictors of burnout on the reduced personal accomplishment subscale of the MBI [81].

Patient care was also affected by high rates of burnout among healthcare providers [79, 80, 101]. For example, among healthcare providers in Malawi, burnout was associated with self-reported suboptimal patient care (OR = 3.22, 95% CI: 2.11–4.90; *p* < 0.0001). Additional factors in the work environment associated with burnout include nursing hierarchy and poor wages [81], staffing issues [79], difficulty communicating with patients [62], organizational complaints [89], job insecurity [97], and intention to quit [88].

Among healthcare providers, burnout is also associated with interpersonal and professional conflicts. Burnout is associated with high level of doctor/doctor conflict [58], doctor/nurse conflict [81], work/family conflict [69], and interpersonal conflict in general [89]. Among doctors in Nigeria (*N* = 204), those who did not report doctor/doctor conflict were less likely to have burnout on the depersonalization subscale of the MBI (OR = 0.36; 95% CI = 0.17–0.76) [58]. Among nurses in Nigeria (*N* = 270), doctor/nurse conflict was a predictor of burnout on the MBI emotional exhaustion subscale (OR = 3.1, 95% CI: 1.9–6.3) and on the depersonalization subscale (OR = 3.4, 95% CI: 2.2–7.6) [81]. Among nurses from public hospitals in Ghana (*N* = 134), work-to-family and family-to-work conflict accounted for 20% of the variance in burnout [69].

Experiences of stress and emotional distress were associated with increased odds of burnout. Among junior physicians in South Africa (*N* = 126), the Physician Stress Inventory (PSI) score was significantly higher among participants with burnout (*p* < 0.001) [62]. Physicians undergoing residency training in Nigeria who reported emotional distress were more likely to report burnout (*p* < 0.001) [58]. In a population of nurses in South Africa (*N* = 122), emotional management and emotional control, as measured by the Swinburne University Emotional Intelligence test, were associated with self-reported stress and burnout subscales (*p* < 0.01). Emotional intelligence was a moderator of the relationship between stress and burnout, explaining 59.5% of the variance in the emotional exhaustion and 23.9% of the variance in the depersonalization subscale of burnout [76]. Among nurses at a hospital in Nigeria, use of emotion-focused coping strategies was positively associated with the MBI burnout subscales of emotional exhaustion (β = 0.32, *p* = 0.01) and depersonalization (β = 0.18, *p* = 0.01) [86].

Lastly, social support was found to be protective against burnout among healthcare providers [58, 79, 80, 91, 97]. Specifically, among physicians in Nigeria, adequate support from management (OR = 0.45; 95% CI:0.22–0.90) were protective from burnout on the MBI subscale of reduced personal accomplishment [59]. Among nurses in Kenya, Tanzania, and Uganda (*N* = 309), lower social support from colleagues was associated with increased burnout on the MBI subscale of higher emotional exhaustion (β = − 0.15, *p* < 0.05) [91].

### Burnout intervention programs

Programs aimed at coping with burnout are sparse. Only two studies, in combined populations of healthcare workers, examined burnout-related interventions [104, 105]. The Support, Train and Empower Managers (STEM) study was designed to implement a support intervention and measure the impact on healthcare workers in Mozambique [105]. At baseline, 67.1% of healthcare workers reported low, 15.9% moderate, and 17.1% high burnout on the MBI. After the intervention, 71.1% reported low, 17.8% moderate, and 11.1% high burnout. However, the authors found no statistically significant differences in emotional exhaustion from baseline to post-intervention for any intervention groups. Job satisfaction, emotional exhaustion and work engagement also showed no significant differences between baseline and post-intervention [105]. Ledikwe and colleagues examined healthcare workers at a public health facility in Botswana (*N* = 1348) after participation in Botswana’s Workplace Wellness Program (WWP) [104]. Job satisfaction, assessed by the Job In General Scale, was significantly higher for healthcare workers who participated in 7 or more activities in the WWP compared to those who did not participate in any activities (*p* = 0.004). Healthcare workers who participated in seven or more WWP activities had significantly higher scores on the Job Descriptive Index subscales related to satisfaction with work, supervision, promotion opportunities and pay, with the highest levels found among those participating in seven or more WWP activities (*p* < 0.05). Additionally, stress levels (*p* = 0.006), measured on the Stress in General scale, and exhaustion (*p* < 0.001), measured on the MBI, were significantly lower among those with high participation in WWP activities [104].

## Discussion

Burnout is common among physicians, nurses, and other healthcare providers in sub-Saharan Africa with prevalence estimates ranging from 40 to 80%. Our findings can be compared to other systematic reviews of burnout among healthcare providers. Among physicians in China (*N* = 9302 participants from 11 studies), burnout prevalence ranged from 66.5–87.8% [39]. Among healthcare providers in Arab countries (*N* = 4108 from 19 studies), high burnout prevalence was estimated in the MBI subscales of emotional exhaustion (20.0–81.0%), depersonalization (9.2–80.0%), and personal accomplishment (13.3–85.8%) [43]. In a recent review (*N* = 109,628 from 182 studies), 67% of physicians reported burnout [124]. Finally, high prevalence of burnout has been reported among emergency room (26%) [125] and pediatric nurses (21–39%) [126].

In sub-Saharan Africa, the highest levels of burnout were recorded among nurses, although all healthcare providers reported high levels of burnout. High levels of burnout were associated with unfavorable work conditions, high job demands, and low job satisfaction. Studies in sub-Saharan Africa support other studies among healthcare providers that have shown burnout is more common among women [43, 127, 128], those of younger age [129], and those with less support or resources to manage workloads [39, 82, 130–132].

### Limitations of current studies

The majority of studies assessed burnout using the MBI. Among those that used the MBI, burnout scores were variously reported as (1) percentage of participants with high burnout on each subscale [58, 72, 81, 87, 99, 106, 116], (2) percentage of participants with high burnout on each subscale and total score [55, 66, 101] (3) percentage of participants with high total burnout [91, 105], (4) percentage of participants with high burnout on emotional exhaustion subscale only [71], (5) total and individual burnout as a continuous scores [59], (6) total burnout as continuous score [69, 78, 110], (7) individual burnout as continuous score [60, 64, 70, 73, 75, 76, 82, 88, 90, 104, 108, 109, 112, 115, 117], or (8) both individual burnout as continuous sores and percentages of participants with high burnout [56, 57, 61, 77, 96]. Furthermore, included studies used four different versions of the MBI to assess burnout including the MBI, MBI-HSS, Abbreviated MBI, and MBI-GS. This introduced difficulty in directly comparing burnout rates between different populations of healthcare providers. Concerns have been raised about how the MBI operationalizes burnout [133]. Despite evidence documenting increasing burnout in sub-Saharan Africa, none of the studies reviewed discuss the conceptual definitions of burnout from a theoretical perspective. Most used the MBI and adopted the three domains of burnout from the MBI scale. Additionally, prior studies have not validated the MBI in healthcare workers in sub-Saharan Africa, and there may be different cultural interpretations of questions related to the construct of burnout. Although it’s difficult to quantitatively compare across populations due to variation in how burnout was defined, burnout prevalence reported using MBI subscales ranged from 12.5–65.2% on emotional exhaustion, 5–57.8% on depersonalization, and 25–85.1% on reduced personal accomplishment. On other instruments, burnout prevalence was 52.4% using one question (‘Do you ever feel so emotionally exhausted that you feel negative about yourself and about your job and lose the feeling of concern for your patients’) [62], 95.4% on the Compassion Fatigue Self-Test [102], 51% using an occupational burnout scale [103], and 63.75% using the ProQOL burnout subscale [118]. Given the variability that exists in assessing burnout in different contexts and with different instruments, there is a need to design studies aimed at evaluating the reliability of various burnout screening instruments cross-culturally.

There are additional limitations to the current studies. A total of 18 studies examined burnout among combined populations of healthcare workers in sub-Saharan Africa (Table 3). These populations include all workers in a clinic or hospital setting, who may have highly variable job responsibilities and workload. In addition, the majority of studies were cross-sectional. Only two studies examined burnout-related interventions in sub-Saharan African populations. Additionally, among the included studies, sample sizes were relatively small and study quality varied widely (Additional file 1: Table S3-S6).

Future studies need to address the drivers of burnout among healthcare providers in sub-Saharan Africa. Although burnout among healthcare providers has been associated with violence against healthcare providers [134, 135]; few studies have examined violence [99] and secondary traumatic stress [114] in sub-Saharan Africa. Performing longitudinal assessments of burnout along with measurements of mood, substance use, suicidality, cognition, performance and quality of life will add to our understanding of the burnout syndrome and its consequences. Efforts should also include utilizing consistent measures of burnout with an instrument validated in specific geographical and cultural contexts.

## Conclusions

Burnout has received a great deal of attention in high-income countries with awareness and intervention programs designed to cope with burnout symptoms. In the United States, a recent report recommends addressing physician burnout by improving physician access to mental health services, improving the usability of electronic medical records, and appointing wellness officials to assess and improve burnout interventions at their institutions [136]. However, burnout among healthcare providers is not only a crisis in high-income countries [124]. It is a significant problem in low and middle income countries as well. Programs aimed at raising awareness, promoting well-being and prevention, and improving coping with burnout symptoms through evidence-based stress management and resilience training in sub-Saharan Africa are needed. Given the ever-increasing burden of major public health threats of communicable and non-communicable diseases in sub-Saharan Africa amidst a dearth of resources and lack of support, along with the adverse health effects of this burden for patients and providers alike, more attention needs to be paid to healthcare provider burnout in low-income settings in Africa and around the world. Additional studies need to address both personal and organizational barriers that increase the risk of burnout among healthcare providers [137]. Individual and structural interventions will need to be combined to effectively reduce burnout among healthcare providers [138]. These interventions should include advocacy for better resource provisions and support for healthcare providers so that healthcare infrastructure and patient care can be improved.

## Additional file

Additional file 1: **Table S1.** Preferred Reporting Items for Systematic Review and Meta-Analyses (PRISMA) guidelines. **Table S2**. Database terms of search. **Table S3.** Quality assessment of studies on burnout among physicians in sub-Saharan Africa (*N* = 12). **Table S4.** Quality assessment of studies on burnout among nurses in sub-Saharan Africa (*N* = 26). **Table S5.** Quality assessment on burnout among healthcare workers in sub-Saharan Africa (*N* = 18). **Table S6.** Quality assessment on burnout among midwives and health professional students in sub-Saharan Africa (*N* = 9). (DOCX 34 kb)

## Data Availability

Not applicable

## Abbreviations

CBI: Copenhagen Burnout Inventory
MBI: Maslach Burnout Inventory
MBI-GS: Maslach Burnout Inventory - General Survey
MBI-HSS: Maslach Burnout Inventory - Human Services Survey
PRISMA: Preferred Reporting Items for Systematic Review and Meta-Analyses
ProQOL: Professional Quality of Life Scale
STEM: Support, Train and Empower Managers
WWP: Workplace Wellness Program
